# Factors associated with the extent of nurses’ involvement in promotion of the nursing profession: a cross-sectional study among nurses working in diverse healthcare settings

**DOI:** 10.1186/s12912-023-01205-z

**Published:** 2023-02-23

**Authors:** Violetta Rozani, Ilya Kagan

**Affiliations:** 1 Department of Nursing, Sackler Faculty of Medicine, The Stanley Steyer School of Health Professions, Tel Aviv University, Tel Aviv, Israel; 2 Ashkelon Academic College, Ashkelon, Israel

**Keywords:** Positive organizational nursing work environment, Promotion of the nursing profession

## Abstract

**Aims and objectives:**

To examine factors associated with promotion of the nursing profession, to the nursing community, other healthcare professionals and the general public, by nurses employed in diverse healthcare settings.

**Background:**

Little is known about the factors that contribute to nurses’ involvement in the promotion of the nursing profession.

**Methods:**

This cross-sectional study comprises a convenience sample of registered nurses (n = 526) with ≥ 3 months’ experience in nursing, who are employed in internal and surgical units, pediatric wards, psychiatric wards or in the community across Israel. Participants completed a self-administered questionnaire addressing socio-demographic, occupational and organizational factors relating to the nursing work environment and to the extent of nurses’ involvement in promotion of the nursing profession. Descriptive statistics, one-way analysis of variance, Pearson’s correlation analysis, and multivariate linear regression were performed.

**Results:**

Nurses in all the surveyed healthcare settings reported relatively low levels of involvement in promotion of the profession, either to the nursing community or to other healthcare professionals and the public. Being a female-nurse was significantly negatively associated with nurses’ involvement in promotion of the nursing profession. In contrast, holding a master’s degree in nursing, having a managerial role, and working in a supportive organizational nursing work environment were the principal factors associated with positive promotion of the nursing profession.

**Conclusion:**

The extent of nurses’ involvement in promotion of the nursing profession mainly depends on occupational factors and a supportive organizational nursing work environment.

**Clinical relevance:**

Healthcare managers may advance nurses’ involvement in promotion of the profession by modifying the organizational nursing work environment. Specifically, we recommend nurse leaders to dedicate efforts to make professional promotion an integral part of a nurse’s role.

**What does this paper contribute to the wider global clinical community?**

- Nurses across all healthcare settings reported relatively low levels of involvement in promotion of the profession, both to the nursing community and to other healthcare professionals and members of the general public.
- Nurses in community care settings scored higher for both nursing work environment and nurses’ involvement in the promotion than nurses working in hospital wards.
- Being a woman was significantly and negatively associated with nurses’ involvement in promotion of the nursing profession.
- Holding master’s degree in nursing, participating in management, and working in a supportive organizational nursing work environment have a positive association with nurses’ involvement in promotion of the nursing profession.
- Healthcare managers should dedicate efforts to make professional promotion an integral part of a nurse’s role.

## Introduction

The World Health Organization (WHO) reports that there are 28 million nurses across the globe, a number that represents 59% of the total number of global health professionals [1]. Nevertheless, ‘Third Global Forum on Human Resources for Health’ estimates that by 2035, the nursing deficit will reach 12.9 million [2, 3]. Although the worldwide pandemic of COVID-19 has improved the popularity of the nursing profession in the public eye [4], it has also led to more burnout among nurses [5, 6], which may increase turnover. Consequently, this shortage is becoming an international problem and one of the main factors determining future world health policy [1, 6, 7]. It has been suggested that building an appropriate public image of the nursing profession may provide a solution to the nursing shortage [1, 8–10]. Several studies show that the public image of the nursing profession is diverse and incongruous [11] and partly self-created by nurses due to their invisibility and their lack of engagement with public discourse and relations [12]. Despite the crucial role that nurses could play in promotion of the image of the nursing, studies concluded that nurses do not generally prioritize the image of their profession, and do not put enough effort into creating and maintaining their persona [9, 10]. Moreover, nurses typically demonstrate little tendency to self-aggrandizement [13, 14] and do not engage in presenting nurses to the public [15–17]. This may include a lack of involvement in policy forums, reaching out to local people and speaking at public gatherings, providing policy-related information to policymakers, advocating for nurses, participating in meetings and discussions, seeking recommendations for other nurses, regulating nursing practice and education, or communicating the developed policies to other nurses [12]. There is little information about the factors associated with the extent to which individual nurses are involved in promotion of the nursing profession. Several studies have highlighted the organizational nursing work environment as a key factor that may influence nursing involvement in promotion of the profession [11, 18–20]. There is now accumulated evidence that a supportive nursing work environment leads to a high quality of nursing care and improves outcomes, for both nurses and patients [21, 22]. This supportive work environment may be manifested as positive organizational characteristics e.g. adequate staffing, flexible scheduling, nurse-physician communication, supportive leadership, and career advancement opportunities. Positive workplace provides nurses with the opportunity to acquire professional experience [21] and enable them to use their knowledge and expertise to provide high-quality care [21–23]. In addition, there is empirical evidence that nurses are more likely to be satisfied with their jobs and are less likely to experience burnout if they feel comfortable within the organization [24]. Through professional interactions with other nurses and colleagues in the workplace, they shape their professional perceptions and self-esteem, which may contribute to their personal growth and provide the motivation to promote the nursing profession to the nursing community, other healthcare professionals and the public. Thus, a broader understanding of, and more information about, the organizational nursing work environment may guide nurse and healthcare administrators in developing strategies to engage nurses in the promotion of the nursing profession. This study was, therefore, designed to redress the lack of relevant information available, and to examine the associations between the socio-demographic, occupational and organizational nursing work environment factors and nurses’ involvement in the promotion of the nursing profession by nurses working in diverse healthcare settings.

## Methods

### Sample

This cross-sectional study involves a convenience sample of 526 registered nurses with at least 3 months of nursing experience (inclusion criterion) and employed in various healthcare settings throughout Israel.

### Power analysis

We used the G*Power program to calculate the sample size for linear multiple regression. The calculation used an effect size of 0.10, and α = 0.05, considering seven predictors in the model. The results indicated that 226 participants will provide 95% power. Since, the study population comprises a convenience sampling, the final study population comprised 526 registered nurses.

### Research instrument

The study used a self-administered structured questionnaire with three parts.

*The first part* examines the nurse’s socio-demographic and occupational characteristics (8 items): sex, age, country of birth, type of healthcare organization, professional qualification, position, experience in nursing, and type of employment.

*The second part* examined Nursing Work Environment using the Hebrew version comprising 20 items [18] of the Revised Nursing Work Index (NWI-R) [25]. Examples of the Nursing Work Environment items are: “Staff nurses are involved in the internal governance of the hospital,” “There is career development/clinical ladder opportunity”, “A nurse manager backs up the nursing staff in decision-making, even if the conflict is with a physician,” Nurses were asked to rank their level of agreement on a scale from 1 (strongly disagree) to 5 (strongly agree). The overall score was represented by the mean. The higher the overall score, the more the work environment is perceived as supporting and promoting nurses and the nursing profession. Cronbach’s alpha for this part of the tool was 0.91.

*The third part* examined the level of nurses’ involvement in promotion of the nursing profession by health- and nursing-related activities, targeting the nursing community, other healthcare professionals and the public. This used a 17-item questionnaire developed and validated in a previous study by Kagan et al., [19]. Examples of nursing-related activities, targeting the nursing community items are: “Holding talks aimed at marketing the profession to nurses from other fields” or “Lecturing to nursing students”. Nursing-related activities, targeting the other healthcare professionals and members of the general public, for example: “Taking part in professional association activities” or “Introducing topics and academic projects to medical or paramedical colleagues”. Nurses were asked to rank the frequency of their involvement in promotion of the nursing profession over the last 2 years, on a scale ranging from 1 (never) to 6 (weekly). The overall score was represented by the mean, with a higher score, reflecting more nurse involvement. Cronbach’s alpha was 0.90.

### Procedure

Data were collected over three months, from April to June 2017.

### Data analyses

Descriptive statistical analyses to describe the general characteristics of the study population summarized the continuous variables as the minimum, maximum, mean (standard deviation), and median. Categorical variables presented as frequencies with percentages. Univariate analyses included bivariate Pearson correlation, and a one-way analysis of variance (ANOVA) followed by Tukey’s post hoc tests to determine the healthcare settings in which work environment and professional promotion by nurses differed significantly. Only the covariates found to be significant for promotion of the nursing profession in univariate analysis were included in the final multivariate analysis. A multiple regression model was used to assess the effects of the independent variables on the prediction of nurses’ involvement in promotion of the nursing profession. Additional statistical models were stratified by the type of healthcare setting. The level of significance was set at a *p*-value of 0.05. The Statistical Package for the Social Sciences version 27 (SPSS Inc., Chicago, Illinois) was used for all data analyses.

## Results

### General characteristics of the study population

The majority of nurses were women (84.6%), with a mean age of 41.5 ± 10.6 years, and a median working experience in nursing of 13.0 years (range 0.33-48.0 years). Slightly more than half the nurses were born in Israel (52.3%), and a fifth of them (22.5%) hold a master’s degree in nursing. Most of the nurses (59.7%) were employed full time as staff nurses (70.3%) and employed at internal medicine and surgical units (42.0%). Socio-demographic and occupational characteristics of the nurses are shown in Table 1.

**Table 1:** General characteristics of the study population (N = 526)

Characteristics	
**Age (years)**	
Mean (SD) Median (Q25; Q75) Min-Max	41.53 (10.58) 40.00 (32.75;49.00) 23.00–79.00
**Age- groups** ***n*** **(%)**	
21–30 years 31–40 years 41–50 years 51–60 years 61–70 years 71 + years	85 (16.2) 180 (34.2) 148 (28.1) 92 (17.5) 17 (3.2) 4 (0.8)
**Sex N (%)**	
Women Men	445 (84.6) 81 (15.4)
**Origin N (%)**	
Born in Israel Born in former Soviet Union Born in other countries Missing	275 (52.3) 469 (40.7) 36 (6.8) 1 (0.2)
**Working experience (years)**	
Mean (SD) Median Min-Max	14.9 (10.8) 13.00 0.33-48.0
**Professional qualification N (%)**	
Registered nurse Registered nurse with BA Registered nurse with MA Missing	109 (20.7) 298 (56.7) 117 (22.5) 2 (0.4)
**Position N (%)**	
Management role Staff nurse Missing	299 (28.9) 370 (70.3) 4 (0.8)
**Scope of employment N (%)**	
100% 76–90% 51–75% 33–50% Missing	314 (59.7) 96 (18.3) 100 (19.0) 13 (2.5) 1 (0.2)
**Type of healthcare settings N (%)**	
General Pediatric Psychiatric Community	221 (42.0) 107 (20.3) 73 (13.9) 125 (23.8)
**Nursing work environment**	
Mean (SD) Median Min-Max	3.39 (0.67) 3.40 1.0–5.0
**Promotion of the nursing profession by nurses**	
Mean (SD) Median Min-Max	2.11 (0.84) 1.88 1.0–6.0

Nurses ranked their work environment as supportive, with the mean score of 3.39 ± 0.67 (on a scale of 1–5). The Highest score was observed in the community setting (M = 3.59 ± 0.67); and a significant difference between the healthcare settings [F (3,522) = 4.748; *p* < .001] was observed. A post hoc Tukey’s HSD Test revealed significantly differences between the mean score of nursing work environment in community and internal medicine and surgical units (*p* = .007) and pediatric wards (*p = .006*).

The mean scores for nurses’ involvement in promotion of the nursing profession were relatively low with a value of 2.11 ± 0.84 (on a scale of 1–6). This means that over the past two years, nurses were involved in promotion of the nursing profession to the nursing community, or to other healthcare professionals, and the general public, maybe once a year. The highest score was also found in the community settings (M = 2.16 ± 0.81); and no significant difference was found between the settings [F (3,522) = 1.601; *p* = .188]. The mean scores of nursing work environment and nurses’ involvement in promotion of the nursing profession in four healthcare settings are presented in Table 2.

**Table 2:** Comparison of mean scores of nursing work environment and nurses’ involvement in the promotion of the nursing profession between the four healthcare settings

Healthcare settings	*n*	Nursing work environment			Nurses’ involvement in the promotion of the nursing profession		
		M (SD)	*F*-statistic (df1, df2)^a^	*p-* value ^a^	M (SD)	*F*-statistic (df1, df2)^a^	*p-* value ^*a*^
Internal medicine and surgical units	221	3.34 (0.65)	4.748 (3,522)	.003^b^	2.13 (0.87)	1.601 (3,522)	.188^c^
Pediatric wards	107	3.25 (0.70)			1.76 (0.83)		
Psychiatric wards	73	3.34 (0.63)			2.00 (0.83)		
Community	125	3.59 (0.67)			2.16 (0.81)		

Abbreviation: M (SD), mean (standard deviation)

^a^ One-way ANOVA

^b^Post-hoc analysis with Tukey’s HSD Test shows significant/borderline difference between community and (a) internal medicine and surgical units (*p* = .007); (b) pediatric (*p* = .006) and (c) psychiatric (p = .068) healthcare settings

^c^Post-hoc analysis with Tukey’s HSD Test shows no significant difference between healthcare settings.

The association between age and promotion of the nursing profession by nurses was not significant (*r* = .063; *p* = .149). However, nurses in full time employment demonstrated a significantly [*t*(524)=-2.1, *p* = .035] higher mean score of the promotion of the nursing profession (*M* = 2.18, *SD* = 0.89) than nurses who worked part time (*M* = 2.01, *SD* = 0.75).

In addition, there was a positive correlation between nursing work environment and the nurses’ involvement in the promotion of the nursing profession only among nurses employed in internal medicine and surgical units (r = .200; p = .002), or in pediatric wards (r = .396; p ≤ .001). It therefore appears that nurses who perceive their work environment as supportive, are more involved in promotion of the nursing profession.

### Estimation of the level of nurses’ involvement in promotion of the nursing profession

Table 3 A presents the estimates from a multivariate regression model designed to predict the level of the involvement of the entire study population in promotion of the nursing profession. Being a female nurse was significantly and negatively associated with the level of involvement in promotion of the nursing profession (β = − 0.091; *p* = .028). Conversely, holding a master’s degree in nursing, having a managerial role, and the supportive nursing work environment were significantly and positively associated with the level of nurses’ involvement in promotion of the nursing profession (β = 0.094, β = 0.269, and β = 0.190 respectively; *p* for all ≤ 0.026). Stratified analysis by healthcare settings (Table 3B) revealed that different independent variables were significantly associated with the level of nurses’ involvement in promotion of the nursing profession. The models could provide 12%, 29%, 14%, and 12% explanation of the variance respectively for the level of nurses’ involvement in promotion of the nursing profession in internal medicine and surgical units, pediatric wards, psychiatric wards, and in the community. Among nurses employed in internal medicine and surgical units, a managerial role and organizational work environment were significantly and positively associated with the level of nurses’ involvement in promotion of the nursing profession (β = 0.206; *p* = .004; β = 0.159; *p* = .016, respectively). Among nurses employed in pediatric wards, a managerial role (β = 0.277; *p* = .003) and a supportive organizational work environment (β = 0.326; *p* < .001) were significantly and positively associated with the level of nurses’ involvement in promotion of the nursing profession. Interestingly, there were no significant associations between independent variables and the level of nurses’ involvement in promotion of the nursing profession among nurses employed in psychiatric wards. However, among nurses employed in the community, holding a master’s degree in nursing was significantly and positively associated with the level of nurses’ involvement in promotion of the nursing profession (β = 0.356; *p < .001*).

**Table 3:** Regression coefficients from the multivariate linear regression model designed to estimate of the level of nurses’ involvement in promotion of the nursing profession *A. The total study population (n = 526)*

Variables				The total study population *n* = 526						
				Standardized Beta (β)			*p*- value			
Sex (women)				− 0.091			0.028			
Holding master degree in Nursing				0.094			0.026			
Working experience				0.001			0.974			
Position (Manager nurse)				0.269			< 0.001			
Organizational Work Environment				0.190			< 0.001			
*B. Stratified study population by type of healthcare setting (n=526)*										
Variables	**Internal medicine and surgical units** ***n*** **= 221**		**Pediatric wards** ***n*** **= 107**			**Psychiatric wards** ***n*** **= 73**			**Community** ***n*** **= 125**	
	Standardized Beta (β)	*p- value*	Standardized Beta (β)		*p- value*	Standardized Beta (β)		*p- value*	Standardized Beta (β)	*p- value*
Sex (women)	− 0.118	0.068	− 0.116		0.198	0.145		0.286	0.017	0.842
Holding master degree in Nursing	0.092	0.164	0.138		0.108	0.178		0.155	0.356	< 0.001
Working experience	0.002	0.977	0.086		0.332	− 0.126		0.318	0.017	0.541
Position (Manager nurse)	0.206	0.004	0.277		0.003	0.125		0.380	0.019	0.840
Organizational Work Environment	0.159	0.016	0.326		< 0.001	0.196		0.094	0.126	0.184

## Discussion

This study was designed to investigate the factors related to nurses’ involvement in the promotion of the nursing profession towards the nursing community, or to other healthcare professionals and the general public. To our knowledge, this the first study to investigate such associations among nurses working in various departments including internal medicine and surgery, pediatrics, psychiatry, or in the community. One of the key findings of the study is that nurses in community care settings exhibited a higher score in both nursing work environment and nurses’ involvement in the promotion scores than nurses working in hospital wards (surgical, internal, pediatric, and psychiatric). This may be due, in part, to the growing roles of nurses in community settings in Israel. These include health promotion, coordination and disease prevention, and management with other healthcare professions. Combined with a broad professional expertise and patient-centric perspective, this makes nurses a precious resource with increased responsibilities for patients, and positions them for greater responsibility and authority [26, 27]. In contrast, nurses in the hospital wards still tend to play the more traditional role of nursing as “handmaiden to medicine” and their autonomy is consequently more limited [27].

The results revealed that nurses across all healthcare settings reported minimal levels of involvement in promotion of the profession. Of note, there were no significant differences in the scores obtained by the four healthcare settings surveyed. Our results are in line with the findings of previous studies [18, 19, 28]. Similarly, an integrative review addressing involvement, and the impact of nurses’ involvement in politics and policy making over the past two decades, revealed that nurses contributed little to politics and policy making [12]. Possible explanations for this low to moderate involvement include a weak feminine nursing image, a feeling of powerlessness with respect to participating in and influencing multidisciplinary decision making, marginalization of the nursing profession, an overdominance of men (particularly physicians) in the area, and the suggestion that nurses lack the confidence, mentorship, and skills to build a positive image of the nursing profession [12, 14, 29]. Alternative explanations for the paucity of promotion might be that nurses feel more confident when interacting with other nurses [19], or might reflect the lack of policy and programs for promoting the status of the profession at an organizational or national level [12, 19, 28].

In our study, nurses ranked their work environment as supportive but there were significant differences in the mean score of nursing work environment recorded in the different healthcare settings. Previous studies have also reported significant differences in nursing work environment [30–32] and the authors concluded that the unique work environment reflects the style of leadership [32, 33], medical complexity and multidisciplinary contact [30], and organizational climate [34] of the specific unit.

Our study also revealed that being a female nurse is significantly and negatively associated with nurses’ involvement in promotion of the nursing profession. Hussien & Fekry [28] believed that this might be due to the traditional feminine family responsibilities, which leave no extra time after work hours for promotional activities. Another option is that female nurses still suffer from gender stereotypes or expectations [9, 11], such as working at the patient’s bedside and performing repetitive and routine tasks as the doctor’s (men) handmaiden. Paradoxically, this image is partly self-perpetuated by nurses because of their invisibility and lack of public engagement [11]. Women-nurses consequently perceive themselves as lacking influence and autonomy and tend not to take initiatives or avail themselves of opportunities to participate in institutional policy making or to participate in policy forums [12]. Nevertheless, nurses in full time employment scored significantly higher on promotion of the nursing profession than nurses in part time employment. Having found no other research reports on this finding, we hypothesize that full time nurses have more opportunities for professional interactions with other colleagues (nurses and other health professional staff), and consequently more professional opportunities.

Our findings also support previous reports [18] that holding master’s degree in nursing is significantly and positively associated with promotion of the nursing profession. Traditionally, the courses in nursing and other science disciplines making up a master’s program, provide advanced theoretical knowledge, assessment skills, tools for role and leadership development, advanced clinical practice in a selected specialization, and the opportunity to critique and apply nursing theory and research as a scientific basis for nursing practice [35]. Consequently, the graduate nursing curriculum is designed to prepare confident and competent leaders for advanced roles such as clinical nurse specialists, nurse practitioners, administrators, teachers, and consultants. Such nurses not only have the knowledge, professional values, skills, and tools required to promote their profession, but also the belief in, and awareness of, the need and importance of promoting nursing to their own community and to others.

A significant and positive association was also found between holding a managerial position and the level of promotion nursing profession by nurses. As part of their job description, nurses in managerial positions are responsible for public relations, marketing, and information technology [19, 36]. Their higher awareness of and commitment to public relations and nursing-wide issues could explain their greater contribution to promotion or marketing of the profession as compared to staff nurses.

In accordance with the findings of Hazanov et al., [18], our results reveal a significant association between nursing work environment and promotion of the nursing profession by nurses. Previous studies reported that a positive work environment was associated with reduced burnout [37], higher organizational commitment, work autonomy, work performance, job satisfaction, and lower job stress [38], as well as a low turnover intention [38, 39]. Our results therefore support the previous suggestions that work environment plays a crucial role in enabling nurse to reach their full potential [22].

In contrast to the association of seniority and promotion of the nursing profession by nurses reported by Kagan et al., [19], we found a null association between these variables.

Our study provides additional information about factors associated with the promotion of nursing profession by nurses, through stratified analysis in different healthcare settings. We hypothesize that the disparities in the associations between independent variables and promotion of the nursing profession across the four healthcare settings surveyed, may be due to factors such as burnout [37], lack of local, organizational, and national policies [12, 19, 28] or leadership [40, 41], job satisfaction 19,28], professional image [11, 18, 19, 28], or gender stereotypes [9, 11, 28]. Since data addressing these factors were not collected in this study, we call for further research to identify the mechanisms by which these factors may affect nurses’ involvement in the promotion of the nursing profession.

Certain limitations of this study should be recognized. First, the data collected in 2017 and were self-reported, which might involve recall or report bias among nurses. Second, because this study was cross-sectional and utilized a convenience sample, we are aware of selection bias potential. We also appreciate that there are other dimensions related to the nurses’ work environment that may also affect nurses’ involvement in the promotion of the nursing profession. These may include organizational and national policies, levels of job satisfaction, professional image among others.

## Conclusion

This study provides new information about factors related to involvement in the promotion of the nursing profession by nurses working in internal medicine and surgical units, pediatric or psychiatric wards, and in the community. The results demonstrate that the extent of nurses’ involvement in promotion of the nursing profession depends on socio-demographic, occupational and organizational nursing work environment factors. Improving the organizational work environment and the academic education of nursing healthcare managers may increase nurses’ involvement in promotion of the profession.

### Clinical relevance

Nurse managers may advance nurses’ involvement in promotion of the profession by modifying the organizational nursing work environment. Accordingly, the organizational work environment offers a powerful target for improvement, and such efforts warrant the resources and attention of health care administrators. Specifically, we recommend nurse leaders to leverage the results and to make dedicated efforts to promotion an integral part of a nurse’s role.

## Data Availability

The data that support the findings of this study are available from the corresponding author, [VR], upon reasonable request.
